# Effects of Soil Tillage, Management Practices, and Mulching Film Application on Soil Health and Peanut Yield in a Continuous Cropping System

**DOI:** 10.3389/fmicb.2020.570924

**Published:** 2020-12-23

**Authors:** Dongqing Yang, Yan Liu, Ying Wang, Fang Gao, Jihao Zhao, Ying Li, Xiangdong Li

**Affiliations:** State Key Laboratory of Crop Biology, Agronomy College of Shandong Agricultural University, Tai’an, China

**Keywords:** tillage, green manure, mulching film, bacterial diversity, soil health, yield, peanut

## Abstract

Our objective was to optimize soil management practices to improve soil health to increase peanut (*Arachis hypogaea* L.) yield. We studied the effects of using rotary tillage with mulching film or without [rotary tillage with no mulching (RTNM)], plow tillage with mulching film or without, and green manure with mulching film (GMMF) or without [green manure with no mulching (GMNM)] over 3 years in Tai’an, China. Results showed that compared with RTNM treatment, GMNM and GMMF treatments significantly (*P* < 0.05) increased soil organic carbon, enzymatic activity, and the available nitrogen, phosphorus, and potassium content. The dominant bacterial phyla in the soil across all treatments were Proteobacteria, Acidobacteria, and Actinobacteria. Bacterial richness and diversity in the soil were significantly (*P* < 0.05) enhanced after GMMF and GMNM treatments compared with those after RTNM treatment. The linear discriminant analysis effect size analysis indicated that Chloroflexi abundance in the 0–10 and 10–20 cm soil layers changed significantly (*P* < 0.05) after rotary tillage with mulching film and RTNM treatments, respectively, whereas that of Bacteroidetes changed significantly (*P* < 0.05) in the 0–10 layer after GMNM treatment. The abundance of the *Xanthobacteraceae* family of Proteobacteria in both soil layers changed significantly (*P* < 0.05) after GMNM and GMMF treatments. Redundancy analysis revealed that soil physical (soil bulk density and water content), chemical (soil organic carbon, available nitrogen, phosphorus, and potassium), and biological (soil enzymatic activity and nutrient content) characteristics affect the soil bacterial community. Changed soil quality indices may be favorable for leaf photo-assimilate accumulation. Compared with RTNM treatment, GMNM and GMMF treatments significantly increased photosynthesis rate in the peanut leaf and decreased intercellular carbon dioxide concentration. Our results showed that compared with that after RTNM treatment, the average pod yield after GMMF and GMNM treatments increased by 27.85 and 21.26%, respectively, due to increases in the pods per plant and plant numbers. The highest yield of all treatments was obtained from the GMMF-treated plot, followed by that from the GMNM-treated plots. Thus, taking into consideration the residual pollution caused by plastic films, we propose GMNM as a suitable strategy to improve soil physicochemical and microbial properties and to increase the peanut pod yield.

## Introduction

Peanut (*Arachis hypogaea* L.) is one of the most important oil crops in the world. In particular, China is the largest peanut producer globally, with major peanut production concentrated in the northern region of the country and production levels that have increased substantially over the past few decades (Yao, 2004). However, peanut production currently faces major challenges owing to long-term continuous monoculture, which has confined its yield within certain limits (Li et al., 2014). Continuous cropping could lead to land degradation, which is referred to as the continuous cropping obstacle (Chen et al., 2017) and is associated with numerous factors such as the deterioration of soil structure (Puerta et al., 2018), a decline in soil fertility (Wacal et al., 2019), and changes in the soil microbial community structure (Chen et al., 2018). Such changes could, in turn, decrease plant development and reduce crop yield. Therefore, the maintenance and improvement of soil quality in peanut continuous cropping systems is critical for sustainable peanut production and the environment.

Soil quality indicators consist of a range of physical, chemical, and biological characteristics linked to key soil ecosystem functions, such as nutrient cycling, soil structure and stability, and soil microbial biodiversity (Muñoz-Rojas, 2018). In recent years, conservation tillage practices, such as reduced tillage and mulch tillage, have been considered to preserve soil health, enhance plant growth, and conserve the environment (Busari et al., 2015). Such outcomes could be attributed to an enhanced nutrient status and an altered soil bacterial community structure (Wang et al., 2016) brought about by conservation tillage. Rotary tillage (RT) is a reduced tillage management practice and is used to mitigate soil erosion and soil organic carbon (SOC) losses in North China (Tian et al., 2016). However, conservation tillage practices could increase soil penetration resistance and strength compared with moldboard plowing (Bogunovic et al., 2018), which makes it difficult for peanut pegs to penetrate the soil surface and leads to a decrease in pod numbers per plant (Haro et al., 2008). Although conventional plowing tillage (PT) could decrease surface soil bulk density (SBD), it would alleviate soil compaction stress and increase available nitrogen (AN), phosphorus (AP), and potassium (AK) concentrations, which would increase crop yield (Shen P. et al., 2016). Therefore, the selection of an appropriate soil tillage practice that creates suitable soil conditions could be an appropriate agronomic management strategy for enhancing peanut yield in a continuous cropping system.

The use of mulching plastic film is a key management practice used to improve spring peanut growth and yield. A previous study has reported that the application of mulching film (MF) increases peanut dry matter accumulation, kernel weight, and pods per plant significantly, resulting in an increase in pod yield (Subrahmaniyan et al., 2018). In addition, MF application reduces soil evaporation rates and increases soil moisture and temperature based on shifting soil condition requirements across different seasons (Sun et al., 2018). Furthermore, plastic film mulching could increase soil bacterial and fungal diversity and richness (Li et al., 2016; Qin et al., 2017), which play crucial roles in soil nutrient cycling (Trivedi et al., 2016). In addition to the positive effects mentioned earlier, the potential negative impact of plastic film pollution on the environment should also be considered (Gao et al., 2019). For example, plastic film results in the release of phthalate esters into the soil, which could be absorbed and accumulated in crops and pose potential risks for human health (Shi et al., 2019). Therefore, other tillage practices and soil management strategies that maintain and improve soil quality and are based on agronomic technologies other than plastic film mulching are required for sustainable peanut production.

Green manure (GM) is commonly sown during fallow periods and applied extensively in agriculture as a strategy for regulating the cycling of soil nutrients such as SOC and N across the soil profile (Sharma et al., 2017). Zhang et al. (2016) suggest that using summer legumes as GM crops is a viable option and an alternative to summer fallow to minimize the risks of N losses and improve subsequent wheat growth. In addition, using Chinese milk vetch (*Astragalus sinicus* L.) as GM significantly increased maize yield by improving soil physicochemical properties, such as alkali solution N, available K, and microbial community diversity (Tao et al., 2017). Growing GM in winter, which would return to the soil in spring, increased microbial abundance in the rice rhizosphere (Zhang X.X. et al., 2017). Based on previous studies, the combination of soil tillage, GM application, and mulching methods may further improve soil structure and nutrient cycling and enhance soil microbial biodiversity. This would improve soil health and address the challenges associated with continuous peanut cropping. Generally, in northern China, the peanut growing period extends from May to October, and the winter fallow is usually bare fallow. We hypothesize that using winter wheat as a cover crop during the winter fallow period and using wheat plants as GM by returning them to the soil at the jointing stage would be a beneficial and cost-effective strategy for enhancing soil health under continuous spring peanut production systems. Therefore, the objectives of the present study were (1) to evaluate the effects of soil tillage and management practices with or without MF on soil physicochemical characteristics and nutrient concentrations; (2) to analyze the effects of different treatments on soil bacterial community structure; and (3) to determine an optimal agronomic management strategy for addressing the challenges associated with the continuous cropping obstacle in peanut production.

## Materials and Methods

### Plant Materials and Growth Conditions

Field experiments were performed over three peanut growing seasons (2016–2018) at the experimental station of Shandong Agricultural University, Tai’an, China (36°09′N, 117°09′E, 128 m above sea level). The region has a warm and semi-humid continental monsoon climate, with an average total annual solar irradiance of 5.08 × 10^6^ kJ cm^–2^, an average annual temperature of 13.7°C, and an average annual rainfall of 631.5 mm. The soil in the study area is classified as Eutric Cambisol, according to the World Reference Base for Soil Resources (2014). The top 20 cm of the soil had a pH of 7.3 and contained 14.56 g kg^–1^ organic matter, 0.75 g kg^–1^ total N, 72.31 mg kg^–1^ AN, 45.62 mg kg^–1^ AP, and 69.35 mg kg^–1^ AK. The SBD was 1.48 g cm^–3^. The peanut cultivar used in the present study was Shanhua 108 (SH108). Seeds were sown in the experimental plots on May 10, 2016; May 8, 2017; and May 8, 2018, and were harvested on October 9, 2016; October 8, 2017; and October 8, 2018, respectively. The peanut was hole-sown at a density of 150,000 hills hm^–2^. Two seeds were planted in each hole, with 25 cm row-spacing and 16.7 cm seed spacing. Triple compound fertilizer [15% N, 15% phosphorus oxide (P_2_O_5_), and 10% potassium oxide (K_2_O); 600 kg ha^–1^] was applied in each treatment before peanut planting. Farm managers used pesticides and herbicides to control disease, pests, and weeds in all treatments.

### Treatments and Experimental Design

The treatments were arranged in a split-plot design with three replications. The main plot included the following: (1) soil conservation tillage management consisting of reduced tillage. Winter fallow period was conventional bare fallow during which no tillage was performed (Supplementary Figures 1A,B) and RT was performed before planting peanut (Supplementary Figure 1C). (2) Tillage management consisted of PT and RT. Plow tillage was performed before the winter fallow period (Supplementary Figure 1D); winter fallow period was bare fallow (Supplementary Figures 1E,F), and RT was performed before planting peanut (Supplementary Figure 1G). (3) GM management consisted of growing winter wheat after harvesting the peanut crop from the previous growing season (Supplementary Figure 1H) and applying the wheat as GM at the jointing stage (Supplementary Figures 1I–K). Wheat cultivar Jimai22 was sown at a density of 2,250,000 plant hm^–2^. No fertilizer was applied during the wheat-growing period. The application rate of GM was based on dry biomass and is illustrated in Supplementary Figure 2. Two subplots were assigned to MF and no mulching film treatments when peanuts were sown. The plastic film was clear and impermeable polyethylene with a thickness of 0.008 mm. The film was removed after the peanut was harvested. A new film was used in the following growing season. Therefore, six treatments were applied and were denoted as follows: (i) rotary tillage with no mulching (RTNM); (ii) rotary tillage with mulching film (RTMF); (iii) plow tillage without mulching film (PTNM); (iv) plow tillage with mulching film (PTMF); (v) green manure with no mulching (GMNM); (vi) green manure with mulching film (GMMF). Each treatment had three replicate plots, and each plot (30 × 2.4 m) was set up in the ridge-furrow fields and consisted of six rows and two ridges.

### Sampling and Analysis

#### Measurement of Soil Bulk Density, Soil Porosity, and Soil Water Content

Soil samples were collected at 0–10 and 10–20 cm depths from three sampling points in each treatment plot at the code 75 stage in the Biologische Bundesanstalt, Bundessortenamt and Chemical (BBCH) scale (Munger et al., 1998). Soil profiles were dug and prepared for sample collection. Undisturbed soil cores were collected using 200 cm^3^ cutting rings at 0–10 and 10–20 cm depths and used to determine SBD, soil porosity, and soil water content (SWC).

#### Measurement of Soil Nutrients and Soil Enzymatic Activity

Three replicates of soil samples were collected using a soil auger (5.0 cm diameter) at 0–10 and 10–20 cm depths from each treatment plot at the code 75 stage in the BBCH scale (Munger et al., 1998), air-dried, sieved through a 2 mm sieve, and used for analyses of SOC, AN, AP, AK, and soil enzymatic activity according to the methods described by Guan (1986) and Bao (2000). The SOC was measured by humid oxidation with potassium dichromate (K_2_Cr_2_O_7_). We measured AN using the modified alkaline hydrolysis diffusion method, AP was obtained by sodium bicarbonate (NaHCO_3_) extraction and then analyzed using the Mo-Sb method with a spectrophotometer, and AK was extracted with ammonium acetate and determined using flame photometry. Soil invertase activity was determined using the 3, 5-dinitrosalicylic acid colorimetric method, whereas urease activity was determined using sodium phenolate and sodium hypochlorite spectrophotometry. Catalase (CAT) activity was determined by back-titrating residual hydrogen peroxide (H_2_O_2_) with potassium permanganate (KMnO_4_).

#### DNA Extraction, Bacterial 16S Ribosomal RNA Gene Polymerase Chain Reaction Amplification, and Illumina Sequencing

Three replicates of soil samples were collected using a soil auger (5.0 cm diameter) at 0–10 and 10–20 cm depths from each treatment plot at the code 75 stage in the BBCH scale (Munger et al., 1998) and stored at −80°C for microbial analyses. Microbial DNA was extracted from 0.5 g soil samples using the DNeasy PowerSoil Pro kit (Qiagen, Hilden, Germany) following the manufacturer’s protocol. Primers F341 (5′-CCTACGGGNGGCWGCAG-3′) and R785 (5′-GACTACHVGGGTATCTAATCC-3′) targeting the V3–V4 region of the 16S ribosomal RNA gene were used for PCR (Klindworth et al., 2013). The PCR assays were performed in a reaction mixture (25 μl) containing 2.5 μl microbial DNA, 0.25 μl of each primer, 12.5 μl 2 × KAPA HiFi HotStart ReadyMix (KAPA Biosystems, Wilmington, MA, United States), and 9.5 μl PCR-grade water. The amplification was performed as follows: 1 cycle of denaturation at 95°C for 3 min; 25 cycles at 95°C for 30 s, annealing at 55°C for 30 s, elongation at 72°C for 30 s, and a final extension at 72°C for 5 min. The products were investigated and checked by electrophoresis in 2% agarose gels. The first round of PCR products was cleaned up using Agencourt AMPure XP beads (Beckman Coulter, CA, United States) according to the manufacturer’s instructions. The second round of PCR assays were performed in a reaction mixture (25 μl) containing 2.5 μl of the first PCR products, 0.25 μl of each primer, 12.5 μl 2 × KAPA HiFi HotStart ReadyMix (KAPA Biosystems, MA, United States), and 9.5 μl PCR-grade water. The amplification was performed as follows: 1 cycle of denaturation at 95°C for 3 min; 8 cycles of 95°C for 30 s, annealing at 55°C for 30 s, elongation at 72°C for 30 s, and a final extension at 72°C for 5 min. The products were investigated and checked using electrophoresis in 2% agarose gels. The selected gel was purified with a QIAquick Gel Extraction kit (Qiagen, Hilden, Germany) according to the manufacturer’s instructions. Before sequencing, the quality of DNA libraries was assessed using a Qubit Fluorometer (Thermo Fisher Scientific, MA, United States), the length distribution of DNA libraries was assessed using a Qseq100 DNA Analyzer (BiOptic Inc., Taiwan, China), and the molarity of the DNA libraries was quantified using the KAPA Library Quantification Kit (KAPA Biosystems, MA, United States). The qualified libraries were sequenced by Beijing Ori-gene Science and Technology Co., Ltd. (Beijing, China) on an Illumina MiSeq platform. The image data were converted to original sequences through Base Calling analysis to form raw data and then merged to tags using FLASH v.1.2.11 (Magoc and Salzberg, 2011). These were then filtered to eliminate the low-quality reads using MOTHUR v.1.35.1 (Schloss et al., 2009) and chimera excluded using UCHIME v.7.0 to obtain clean reads based on the reference Gold database (Edgar et al., 2011; Haas et al., 2011). The clean sequences were clustered into operational taxonomic units based on sequence similarity (97% threshold) by USEARCH (Edgar, 2013). Taxonomy analysis was performed using the RDP v.2.2 classifier based on the SILVA database (Pruesse et al., 2007; Wang et al., 2011). The raw sequencing data have been deposited in Figshare and are available at https://figshare.com/articles/dataset/Sequencing_data/12464396.

#### Measurement of Photosynthetic Parameters

Photosynthetic parameters, including photosynthesis rate (P_*N*_), intercellular carbon dioxide concentration (C_*i*_), transpiration rate (T_*r*_), and stomatal conductance (G_*s*_), were measured in all the treatments using a Li-6400 portable photosynthetic system (LI-COR Biosciences Inc., NE, United States) at code 75 stage. All measurements were obtained between 1,000 and 1,100 h. The chamber was equipped with a red/blue light-emitting diode light source, and the photosynthetically active radiation was set at 1,400 μmol m^2^ s^–1^.

#### Measurement of Peanut Yield and Its Components

At maturity (code 99 stage in the BBCH scale), the plants in a 6.67 m^2^ (3.335 × 2 m) area (from which no plants were sampled) were harvested for yield determination. Fifteen representative plants were sampled from each treatment to record the number of pods per plant. All pods harvested from peanut plants were air-dried and weighed. In addition, the shells were peeled to obtain peanut kernel yield and kernels per kilogram.

### Statistical Analysis

Alpha diversity was calculated to estimate richness using Chao1 and the diversity using the Shannon index. For beta diversity, the taxonomic and phylogenetic community comparisons were performed by calculating Bray–Curtis and weighted UniFrac distance matrices. Linear discriminant analysis effect size (LEfSe) was performed based on the absolute abundance of assigned taxa, and linear discriminant analysis > 2.5 was considered a significant difference. The data of peanut yield, soil parameters, and microbial abundance were processed using the DPS v7.05 software (Hangzhou RuiFeng Information Technology Co., Ltd., Hangzhou, China). Significant differences among the means were compared using the least significant difference test at a 5% significance level. Correlation analyses were performed with R v.4.0.2. Redundancy analysis (RDA) was performed with Canoco 5. To avoid overfitting in the regression model due to many explanatory variables, the most discriminating variables for each data set were selected by the “forward selection” procedure of the program during the analysis. Graphs were plotted using SigmaPlot v10 (Systat Software Inc., CA, United States).

## Results

### Effects of Soil Management Practices on Peanut Yield and Its Components

Significant differences were observed in pod yield, pods per plant, pods per kilogram, and plant number among the various treatments (Table 1). As expected, both PTMF and GMMF treatments increased pod yield significantly (*P* < 0.05) by increasing the pods per plant and plant number and decreasing the pods per kilogram, when compared with that in the RTNM treatment. The highest yield among all the treatments was observed in the GMMF-treated plots. For example, compared with that in the RTNM treatment, pod yield was 16.3 and 24.5% higher in the PTNM and GMNM treatments, respectively, in the 2018 season.

**TABLE 1:** Effects of soil tillage and management practices and mulching film on the pod yield and its component.

**Year**	**Treatment**	**Pod yield (kg ha^–1^)**	**Pods per plant**	**Pods per kg**	**Plants number (plants ha^–1^)**
2016	RTNM	4408.1^d^	16.2^e^	567.4^a^	231818.2^e^
	RTMF	4606.2^c^	17.5^d^	562.7^a^	248484.8^d^
	PTNM	4659.1^c^	18.7^c^	526.1^c^	262121.2^b^
	PTMF	4981.3^b^	21.1^b^	521.2^c^	272727.3^b^
	GMNM	4995.1^b^	22.0^b^	549.3^b^	254545.5^c^
	GMMF	5250.5^a^	23.5^a^	544.8^b^	265656.6^a^
2017	RTNM	4565.9^d^	16.1^e^	538.0^a^	222222.2^d^
	RTMF	4698.2^d^	17.7^d^	533.0^a^	239393.9^c^
	PTNM	4778.4^c^	20.5^c^	515.0^b^	268686.9^b^
	PTMF	5095.3^b^	22.3^b^	510.5^b^	277272.7^a^
	GMNM	5171.4^b^	23.8^a^	485.0^c^	267676.8^b^
	GMMF	5435.1^a^	24.4^a^	479.5^c^	278282.8^a^
2018	RTNM	4244.6^e^	14.1^e^	598.0^a^	219191.9^d^
	RTMF	4525.1^d^	18.3^d^	560.0^b^	237878.8^c^
	PTNM	4935.3^c^	21.4^c^	549.3^c^	269697.0^b^
	PTMF	5270.7^b^	23.5^b^	546.0^cd^	276767.7^a^
	GMNM	5285.7^b^	23.8^b^	537.3^de^	266666.7^b^
	GMMF	5606.0^a^	25.6^a^	528.7^e^	281313.1^a^
**ANOVA**					
Year (Y)		299.0**	74.0**	285.9**	7.3**
Practices (P)		4176.9**	2165.2**	235.1**	1200.2**
Mulching (M)		1882.5**	455.2**	31.8**	322.9**
Y × P		110.4**	39.2**	35.7**	44.0**
Y × M		43.4**	20.9**	6.2**	0.3 ns
P × M		6.6**	10.8**	5.1*	12.8**
Y × P × M		22.8**	10.6**	5.3**	0.8 ns

Values followed by different letters within the columns are significantly different at the 0.05 probability level. *Significant at the 0.05 probability level. **Significant at the 0.01 probability level. RTMF, rotary tillage without mulching film; RTMF, rotary tillage with mulching film; PTNM, plow tillage without mulching film; PTMF, plow tillage with mulching film; GMNM, green manure without mulching film; GMMF, green manure with mulching film.

### Effects of Soil Management Practices on Leaf Photosynthetic Performance

**FIGURE 1:**
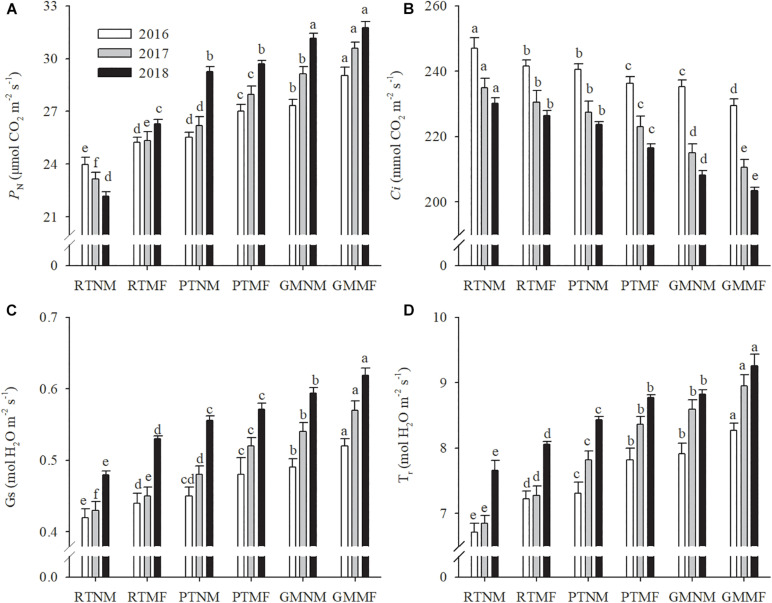
Effects of soil tillage managements and mulching film on leaf photosynthesis rate **(A)**, intercellular carbon dioxide concentration **(B)**, transpiration rate **(C)**, and stomatal conductance **(D)**. Vertical bars represent standard error (SE). Mean values ± SE (*n* = 10). Different letters indicate significant differences among each treatment, *P* < 0.05.

The P_*N*_ in plants subjected to RTNM treatment decreased annually (Figure 1); however, the other treatments exhibited an opposite trend. Intercellular carbon dioxide concentration (C_*i*_) decreased in the three growing seasons. Conversely, stomatal conductance (G_*s*_) exhibited increasing variation between and within treatments, similar to transpiration rate (T_*r*_). Compared with those in peanut plants subjected to RT, both PT and GM soil management practices and MF application increased P_*N*_, Gs, and Tr of peanut plants significantly (*P* < 0.05) and decreased their Ci significantly (*P* < 0.05). On average, P_*N*_ exhibited 5.3 and 4.5% increases in plants subjected to PTMF and GMMF treatments, respectively, when compared with plants under PTNM and GMNM treatments, respectively, across the three growing seasons.

### Effects of Soil Management Practices on Soil Properties

The soil tillage management practices and MF treatments influenced SBD, soil porosity, and SWC significantly (*P* < 0.05) (Table 2). The highest SBD was observed in the RTNM treatment in the 2018 growing season. However, it decreased in the PTMF- and GMMF-treated plots. In addition, the SBD value in the 0–10 cm layer in the 2018 growing season decreased by 13.2 and 17.0% in the PTNM- and GMNM-treated plots, respectively, compared with that in RTNM-treated one. Conversely, RTNM-treated plots had the lowest soil porosity value. Compared with that in the RTMF-treated plots, the soil porosity in the 0–10 cm layer in the 2018 growing season increased by 14.6 and 19.5% in the PTMF- and GMMF-treated plots, respectively, and that in the 10–20 cm layer increased by 15.2 and 20.4%, respectively. Similarly, the lowest SWC was observed in soils under RT. Plow tillage, GM, and MF treatments increased SWC significantly (*P* < 0.05). Compared with that in RTNM-treated soils, PTNM and GMNM treatments increased SWC in the 0–10 cm soil layer by 12.9 and 32.8% on average, respectively, whereas RTMF treatment increased SWC in the 10–20 cm soil layer by 19.7% on an average.

**TABLE 2:** Effects of soil tillage and management practices and mulching film on soil bulk density, porosity, and soil water content in the two layers.

**Year**	**Treatment**	**Soil bulk density**		**Soil porosity**		**Soil water content**	
				
		**0–10 cm**	**10–20 cm**	**0–10 cm**	**10–20 cm**	**0–10 cm**	**10–20 cm**
2016	RTNM	1.49^a^	1.54^a^	43.77^d^	41.73^e^	11.54^f^	12.92^f^
	RTMF	1.47^a^	1.51^b^	44.40^d^	43.16^d^	14.74^e^	16.37^e^
	PTNM	1.42^b^	1.46^c^	46.42^c^	44.97^c^	13.30^d^	15.51^d^
	PTMF	1.38^c^	1.44^c^	48.05^b^	45.64^c^	15.70^c^	16.80^c^
	GMNM	1.35^c^	1.41^d^	48.93^b^	46.82^b^	16.42^b^	17.52^b^
	GMMF	1.31^d^	1.35^e^	50.57^a^	49.03^a^	16.93^a^	18.67^a^
2017	RTNM	1.52^a^	1.57^a^	42.79^f^	40.75^f^	11.21^e^	13.01^e^
	RTMF	1.49^b^	1.51^b^	43.68^e^	42.98^e^	14.91^c^	16.25^cd^
	PTNM	1.41^c^	1.44^c^	46.78^d^	45.64^d^	13.53^d^	15.76^d^
	PTMF	1.36^d^	1.42^d^	48.86^c^	46.53^c^	15.55^b^	16.80^c^
	GMNM	1.33^e^	1.36^e^	49.74^b^	48.57^b^	16.66^a^	17.50^b^
	GMMF	1.31^f^	1.32^f^	50.67^a^	50.35^a^	17.08^a^	18.67^a^
2018	RTNM	1.59^a^	1.61^a^	39.93^f^	39.40^f^	12.90^d^	12.65^e^
	RTMF	1.52^b^	1.54^b^	42.63^e^	42.05^e^	14.40^c^	15.97^d^
	PTNM	1.38^c^	1.42^c^	47.89^d^	46.40^d^	14.20^c^	15.98^d^
	PTMF	1.36^d^	1.37^d^	48.85^c^	48.44^c^	14.55^c^	16.53^c^
	GMNM	1.32^e^	1.35^e^	50.13^b^	49.08^b^	16.22^b^	17.53^b^
	GMMF	1.30^f^	1.31^f^	50.94^a^	50.61^a^	16.99^a^	18.33^a^
**ANOVA**							
Year (Y)		4.1*	13.6**	4.2*	13.5**	0.6	1.7
Practices (P)		1577.7**	1173.6**	1576.5**	1169.5**	728.7**	642.0**
Mulching (M)		159.4**	186.0**	158.3**	185.3**	491.8**	495.1**
Y × P		46.1**	34.3**	46.3**	34.3**	4.8**	0.9
Y × M		0.3	2.4	0.3	2.2	27.2**	2.2
P × M		1.4	4.6*	1.4	4.6*	74.7**	94.5**
Y × P × M		8.3**	2.5	8.3**	2.5	11.9**	0.6

Values followed by different letters within the columns are significantly different at the 0.05 probability level. *Significant at the 0.05 probability level. **Significant at the 0.01 probability level. RTMF, rotary tillage without mulching film; RTMF, rotary tillage with mulching film; PTNM, plow tillage without mulching film; PTMF, plow tillage with mulching film; GMNM, green manure without mulching film; GMMF, green manure with mulching film.

**FIGURE 2:**
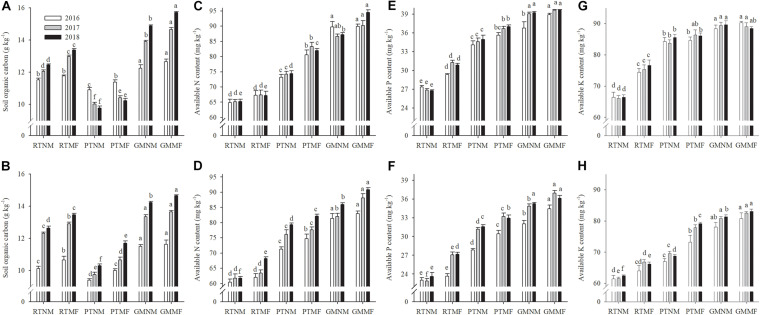
Effects of soil tillage managements and mulching film on soil organic carbon **(A,B)**, available N content **(C,D)**, available P content **(E,F)**, and available K content **(G,H)** in the two layers. Vertical bars represent standard error (SE). Mean values ± SE (*n* = 3). Different letters indicate significant differences among each treatment, *P* < 0.05.

In PT-treated soils, the SOC concentrations were significantly lower compared with those in RT-treated ones (*P* < 0.05) (Figures 2A,B). Conversely, GM application increased SOC concentration in the two soil layers. The GMMF treatment had the highest SOC concentrations in the two soil layers. The AN, AP, and AK concentrations in the two soil layers increased significantly (*P* < 0.05) under PT and GM application practices and MF application (Figures 2C–H). Higher AN values were observed in the 0–10 and 10–20 cm soil layers under GMMF treatment when compared with the AN values in the RTMF. The GMMF treatment had higher AP values in the 0–10 and 10–20 cm soil layers when compared with the AP values in RTMF-treated soils. The variation in AK concentration in the 0–20 cm soil layer was similar to that of AP.

**FIGURE 3:**
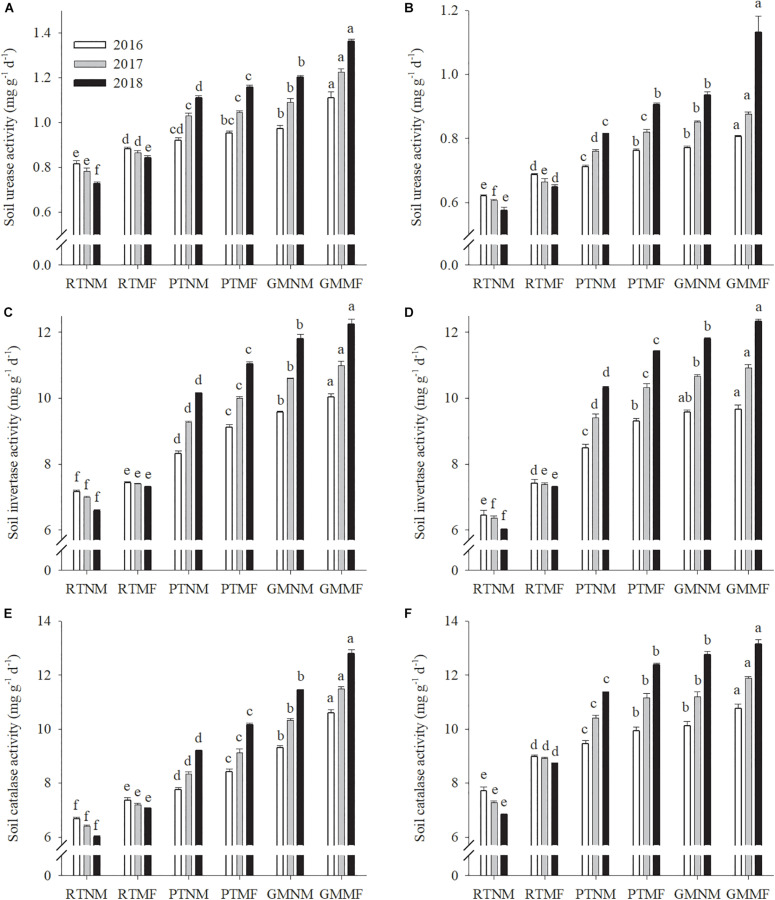
Effects of soil tillage managements and mulching film on soil urease activity **(A,B)**, invertase activity **(C,D)**, and catalase activity **(E,F)** in the two layers. Vertical bars represent standard error (SE). Mean values ± SE (*n* = 3). Different letters indicate significant differences among each treatment, *P* < 0.05.

Reduced soil tillage practices decreased soil urease, invertase, and CAT activity from 2016 to 2018 (Figure 3). In contrast, PT and GM increased the activity of urease, invertase, and CAT annually. The enzyme activity in the soils under mulching treatments was significantly (*P* < 0.05) higher than that in soils under no mulching treatment. The urease activity in the PTMF-treated 0–10 and 10–20 cm soil layers increased significantly (*P* < 0.05) by 37.2 and 39.7%, respectively, and that in GMMF-treated soils increased by 61.6 and 74.4%, respectively, when compared with that in the RTMF-treated soils in the 2018 growing season. Similarly, compared with that in RTMF-treated soils, invertase activity in the PTMF treatment in the 0–10 and 10–20 cm soil layers increased significantly (*P* < 0.05) by 51.2 and 56.3%, respectively, and that in the GMMF treatments in the 0–10 and 10–20 cm soil layers increased significantly (*P* < 0.05) by 67.6 and 68.8%, respectively. Compared with the CAT activity in the GMNM treatment, the CAT activity in the GMMF treatment in the 0–10 and 10–20 cm soil layers increased significantly (*P* < 0.05) by 11.7 and 3.2%, respectively.

### Effects of Soil Management Practices on Soil Bacterial Community Structure

**FIGURE 4:**
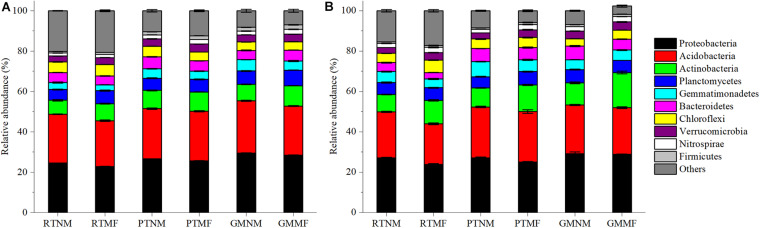
Relative abundance of major taxonomic groups at the phylum level for bacteria in 0–10 cm **(A)** and 10–20 cm soil layer **(B)**. Relative abundance of top 10 phyla is displayed in different colors. Vertical bars represent standard error (SE). Mean values ± SE (*n* = 3).

As illustrated in Figure 4, the relative abundance of the dominant bacterial phyla in the soil was similar among all the treatments. Proteobacteria, Acidobacteria, Actinobacteria, Planctomycetes, Gemmatimonadetes, Bacteroidetes, Chloroflexi, Verrucomicrobia, Nitrospirae, and Firmicutes were the 10 most abundant phyla. Of these, Proteobacteria were the most abundant, followed by Acidobacteria. Plow tillage and GM with or without MF influenced the relative abundance of the phyla significantly (*P* < 0.05) within the two soil layers. However, compared with the RTNM treatment, Proteobacteria abundance in the 0–10 and the 10–20 cm soil layers under RTMF treatment decreased significantly (*P* < 0.05) by 11.8 and 6.9%, respectively. Conversely, the abundance of Actinobacteria and Planctomycetes in the two soil layers increased significantly (*P* < 0.05) after MF treatment. Compared with that under GMNM treatment, the abundance of Actinobacteria in the 0–10 and 10–20 cm soil layers under GMMF treatment increased significantly (*P* < 0.05) by 59.0 and 24.1%, respectively.

**FIGURE 5:**
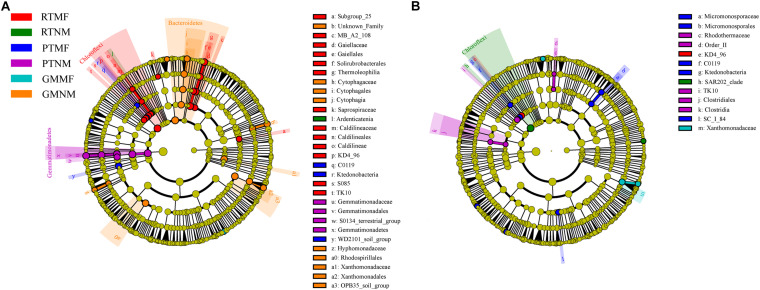
A linear discriminant analysis effect size method identifies the significantly different abundant taxa of bacteria in 0–10 cm **(A)** and 10–20 cm soil layer **(B)** from all treatments.

We used LEfSe analysis from the phylum to genus level to evaluate the significant differences in bacterial abundance among the treatments (Figure 5). Linear discriminant analysis scores higher than 2.5 were applied to determine the specialized bacterial groups enriched in response to soil tillage practices and MF application (Supplementary Figure 3). The LEfSe results showed that the *Ardenticatena* sp. was a biomarker for RTNM treatment. *KD4-96* from Chloroflexi changed in the two soil layers of the RTMF treatment. Gemmatimonadetes were enriched in the 0–10 cm soil layer in the PTNM treatment. *Lysinibacillus* sp. from Firmicutes and *Micromonosporaceae* from Actinobacteria exhibited the greatest changes in the 0–10 and the 10–20 cm layers, respectively, in the PTMF treatment. Bacteroidetes were enriched in the 0–10 cm soil layer under GMNM treatment, whereas the *Byssovorax* and *Xanthomomadaceae* families from Proteobacteria exhibited the greatest changes in the 0–10 and the 10–20 cm soil layers, respectively, under GMMF treatment.

### Effects of Soil Management Practices on Soil Bacteria Alpha and Beta Diversity

Statistically significant differences in soil bacteria richness and diversity were observed under different treatments based on the Chao1 and Shannon diversity indices (Supplementary Figure 4). The Chao1 and Shannon values in the PTNM and GMNM treatments increased significantly (*P* < 0.05) compared with the values in the RTNM treatment.

According to the results, the first axis separates RT from the other treatments, whereas the second axis separates GM and PT for both 0–10 and 10–20 soil layers, suggesting that soil tillage management practices could influence the bacterial community structure (Supplementary Figure 5). Each group included two clusters, indicating that MF application also influenced bacterial community structure.

### Relationships Between Soil Properties and Microbial Community Structure

**FIGURE 6:**
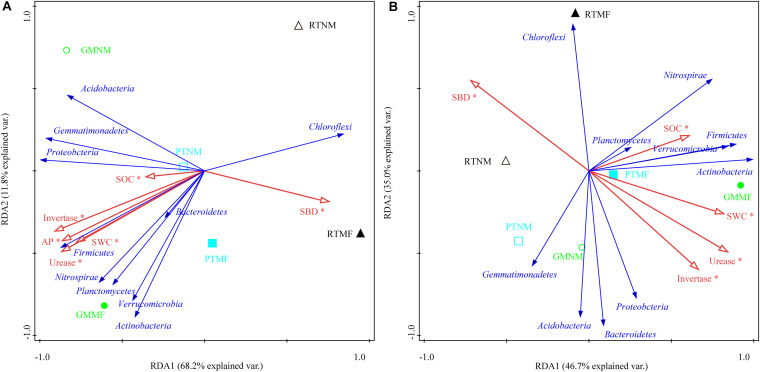
Ordination plots of the results from the redundancy analysis used to explore the relationships between microbial community (blue arrows) and soil physicochemical properties (red arrows) in 0–10 cm **(A)** and 10–20 cm **(B)** soil layers from treatments. SOC, soil organic carbon; soil water content, SWC; SBD, soil bulk density; SP, soil porosity; AN, available N content; AP, available P; AK, available K content; Urease, soil urease activity; Invertase, soil invertase activity; Catalase, soil catalase activity. RT, reduced tillage (triangle), PT, plow tillage (square), GM, green manure (circle). Solid and hollow represent with mulching film (MF) or without MF (NM). *Correlations are significant at *P* < 0.05.

An RDA was used to explore the effect of soil physicochemical properties on the dominant microbial communities (Figure 6). The cumulative variance of contributions reached 80.0% (RDA1 explained 68.2%, and RDA2 explained 11.8%) and 81.7% (RDA1 explained 46.7%, and RDA2 explained 35.0%) in the 0–10 and the 10–20 cm soil layers, respectively. Both SOC and CAT were the major factors that influenced the microbial community structure, which explained 29.22% of the total variance primary factor explaining 68.4% of the variability in the microbial community in the 0–10 cm soil layer, followed by, which explained 64.6% of the variability, followed by SP and urease. Urease was also an explanatory variable (42.9%) for microbial community variability in the 10–20 cm soil layer, followed by SBD, which explained 36.3% of the variability in the microbial community.

## Discussion

### Effects of Soil Management Practices on Yield and Its Components

Conservation tillage practices imply reduced tillage intensity compared with the tillage intensity under moldboard plow inversion and are considered to maintain soil health and enhance plant growth (Reicosky, 2015). However, according to the results of the present study, pod yield in the RT treatment decreased in 2018 compared with the pod yield in the 2016 and 2017 growing seasons. This was because yield components, including pods per plant and plant number, declined gradually over the years (Table 1). Conversely, under PT and GM application, pod yield increased gradually in the growing seasons for the 3 years. It has been reported consistently that enhanced crop yields are associated with changes in photosynthetic characteristics, such as P_*N*_, T_*r*_, and G_*s*_ (Dahal et al., 2014). We found that PT increased P_*N*_ in plants, which increased dry matter accumulation and translocation when compared with that in RT (Shi et al., 2016). In addition, GM application improved chlorophyll content in tobacco leaves (Bilalis et al., 2009). Islam et al. (2018) also reported that GM application increased rice leaf area index and net assimilation rate. Furthermore, MF treatment increased the P_*N*_ of functional wheat leaves at the filling stage (Yang et al., 2018). The results of the present study are consistent with those reported previously, as we found that the P_*N*_, Gs, and T_*r*_ of peanut plants under PT and GM treatments were higher than those under RT; these values under MF were higher than those under the non-MF treatments. Notably, the P_*N*_ under GM treatment increased from 2015 to the 2018 growing seasons. There were no significant differences in P_*N*_ between the GMNM and GMMF treatments in the 2018 growing season (Figure 1), indicating that continuous application of GM could improve leaf photosynthetic performance gradually, and mulching plastic film could be unnecessary after several years of applying GM.

### Effects of Soil Management Practices on Soil Physical and Chemical Properties

Soil bulk density, soil porosity (SP), and SWC are the key soil physical characteristics influenced by soil tillage practices (Celik et al., 2004). In the present study, the values of SBD increased, and those of SP decreased under RTNM treatment from 2015 to 2018 (Supplementary Table 1). Higher SBD and lower SWC values suggest high soil mechanical resistance (Vaz et al., 2001). The PT and GM application combined with or without MF treatments significantly decreased SBD and increased SP and SWC in the two soil layers. This indicates that PT, GM, and MF could reduce soil mechanical impedance, which could enhance root length density and improve root distribution. This would thus increase nutrient uptake and facilitate higher biomass and yield (Guan et al., 2014).

Previous studies have reported that SBD and SWC influence nutrient mineralization and diffusion (Paul et al., 2003; Comerford, 2005). We observed that AN, AP, and AK concentrations increased in the two soil layers under PTNM and GMNM treatments (Figure 2). Further correlation analysis results revealed that SBD was significantly and negatively correlated with AN, AP, and AK concentrations (Supplementary Figure 6). Also, SWC was significantly and positively correlated with AN, AP, and AK concentrations, potentially because PT and GM application combined with or without MF treatments increased soil urease, invertase, and CAT activity compared with that in RTNM-treated soils. The soil enzymes mentioned earlier play pivotal roles in catalyzing reactions associated with organic matter decomposition and nutrient cycling (Das and Varma, 2011). Kabiri et al. (2016) reported that soil tillage increased soil urease and alkaline phosphatase activity, which, in turn, improved soil nutrient cycling (Chen et al., 2019). Mulching increased soil enzymatic activity, which is associated with nutrient dynamics, particularly that of AN (Akhtar et al., 2018). The application of GM as a nutrient source is beneficial to a subsequent crop because of the nutrients that are released when their residues return to the soil (Talgre et al., 2012). In addition, non-legume crops grown as GM or catch crops could effectively decrease nutrient leaching (Couëdel et al., 2018). Correlation analysis results showed that enzyme activity was positively correlated with AN, AP, and AK concentrations. These results suggest that PT, GM, and MF altered soil structure and lead to favorable SBD and SWC that enhanced soil enzyme activity and improved soil nutrient status.

The SOC is an important indicator of soil health and agronomic sustainability due to its influence on soil chemical and biological properties (Reeves, 1997). We observed that PTNM decreased SOC content in the two tested soil layers compared with that in RTNM, which could be because reduced tillage can preserve the labile carbon pools and decrease the supply of mineralizable organic carbon for microbes (Raiesi and Kabiri, 2017). Although using a moldboard plow could loosen the soil and lead to a lower SBD value, it would promote soil carbon oxidation and increase carbon dioxide emissions (Chatskikh and Olesen, 2007). Zhang et al. (2017b) concluded that crops could produce greater root biomass under MF conditions, which would return to the soil and increase SOC. Long-term plastic film mulching would not necessarily lead to a decrease in SOC, as SOC increases in most areas, particularly under poor moisture and temperature conditions (Zhang et al., 2017a). Consistent with these studies, we observed that the 3 years continuous use of MF increased SOC significantly in the 0–10 and 10–20 cm soil layers. The use of a winter cover crop as GM is an important soil management practice for maintaining SOC concentrations (Haque et al., 2015), and winter wheat plants have a higher C:N ratio and biomass (Baggs et al., 2000). The application of wheat straw at a rate of 8 t ha^–1^ could increase SOC concentration (Zhang M. et al., 2017). In the present study, we applied 5.5 t ha^–1^ of winter wheat on average as GM. The GMMF-treated soils had the highest SOC concentration among all treatments, followed by that in GMNM-treated soils.

### Effects of Soil Management Practices on Soil Microbial Community Structure

Bacterial community structure is highly influenced by soil properties and agricultural practices (Shen Y.F. et al., 2016). A previous study has reported that no-tillage management affects the size of soil microbial communities (Tyler, 2019). In the present study, PT and GM application with or without MF altered bacterial abundance at the phylum level compared with that after RT (Figure 4), which could be because microbes are more active under tillage, and soil texture could also influence the effect of tillage on microbial properties (Zuber and Villamil, 2016). Chao1 and Shannon indices are the metrics that are usually used to analyze microbial alpha diversity (Wang et al., 2018). In the present study, GM combined with MF had the highest Chao1 and Shannon index values followed by the other treatments in the following order: GMMF > GMNM > PTMF > PTNM > RTMF > RTNM (Supplementary Figure 4). Soil carbon, N, and P content significantly correlated with Shannon and Chao1 diversity index (Han et al., 2018). In addition, UniFrac is a β-diversity measure that uses phylogenetic information to compare environmental samples (Lozupone et al., 2011). The UniFrac and PLS-DA analysis results in the present study showed that bacterial communities in different soil treatment samples were separated into three groups (Supplementary Figure 5). These results suggest that GM combined with MF changed soil environmental factors, including SOC, SWC, AN, and AP, which resulted in changes in the bacterial richness and diversity in the soil.

In turn, soil microbes play important roles in soil ecosystems, including organic matter and nutrient mineralization and nutrient cycling (Miki et al., 2010; Li et al., 2019). Moreover, Fan et al. (2020) reported that soil biodiversity is essential for supporting crop yield and soil functions. Based on the results of the LEfSe analysis, bacterial species changed significantly among the six treatments in the two soil layers (Figure 5 and Supplementary Figure 3). Chloroflexi changed significantly in the RTMF and RTNM treatments in the 0–10 and 10–20 cm soil layers, respectively, which could be because Chloroflexi are anaerobic and their abundance would increase significantly in hypoxic soil (Šibanc et al., 2014). RT increased SBD and decreased SP (Figure 2), which may result in poor soil permeability. Results of the RDA analysis revealed that Chloroflexi abundance was significantly correlated with SBD (Figure 6). The C0119 class (phylum Chloroflexi) was also common in the two soil layers in the PTMF treatment, which may be because MF may not favor soil breathability. Gemmatimonadetes abundance changed significantly under PTNM treatment in the 0–10 cm soil layer. Sengupta and Dick (2015) reported that plow tillage increased the relative abundance of Gemmatimonadetes significantly. Gemmatimonadetes are widespread in nature and can accumulate polyphosphates and decompose organic matter (Zhang et al., 2003; Takaichi et al., 2010), which could explain why PT decreased SOC but increased AP concentration in the soil. Bacteroidetes are predominant in agricultural systems due to their capacity to exploit bioavailable organic matter rapidly (Huang et al., 2019). As the GM treatment applied a relatively high amount of wheat straw as GM to the soil and increased SOC, Bacteroidetes abundance changed significantly under GMNM treatment in the 0–10 cm layer. The finding that SOC significantly influenced microbial community structure is consistent with the results of Sul et al. (2013), who reported that SOC was the most important factor that effectively explained the differences in microbial community structure. In addition, the abundance of the *Xanthobacteraceae* family of Proteobacteria changed significantly under GMNM and GMMF treatments in the two soil layers. Similarly, Zhou et al. (2016) reported that the *Xanthobacteraceae* family was a microbial group found in abundance during the late stage of wheat straw decomposition. The *Xanthobacteraceae* family exhibited an associative relationship to microbial N (Obermeier et al., 2020). Further, a previous study has reported that *Xanthobacteraceae* can fix N (Lee et al., 2005), which could explain the relatively high AN concentration under GMNM and the GMMF treatments in the two soil layers.

## Conclusion

In the present study, a 3 years reduced tillage treatment increased SOC concentration but decreased AN, AP, and AK concentrations, soil enzyme activity, and soil bacteria richness and diversity. This, in turn, decreased leaf photosynthesis rate and peanut pod yield when compared with conventional tillage treatment. Soil physical (SBD and SWC), chemical (SOC, AN, AP, and AK), and biological (bacterial community) properties were significantly improved by GM and MF application, which resulted in increased photo assimilation and, in turn, peanut pod yield. Overall, the application of GMMF resulted in the highest pod yield, followed by that from the GMNM treatment. Our findings suggest that reduced tillage management is not an appropriate agronomic management strategy for enhancing peanut yield in a continuous cropping system. We propose that GMNM could be a suitable soil management practice for improving soil quality while increasing peanut pod yield, considering the residual pollution associated with plastic films.

## Data Availability Statement

The original contributions presented in the study are publicly available. This data can be found here: https://figshare.com/articles/dataset/Sequencing_data/12464396.

## Author Contributions

DY conceived and designed the experiments, analyzed the data, prepared figures and tables, authored and reviewed drafts of the manuscript, and approved the final draft. YLiu, YW, JZ, and YLi performed the experiments. XL contributed reagents, materials, analysis tools, and authored and reviewed drafts of the manuscript. All authors contributed to the article and approved the submitted version.

## Conflict of Interest

The authors declare that the research was conducted in the absence of any commercial or financial relationships that could be construed as a potential conflict of interest.

## Supplementary Material

Supplementary Figure 1 Different soil tillage and management practices applied to the experimental field. **(A–C)** Represent reduced tillage management. Winter fallow period was bare fallow and no tillage was performed during the period **(A,B)**, and rotary tillage (RT) was adopted before planting peanut **(C)**. **(D–G)** Represent conventional tillage management consisting of plow tillage (PT) and RT. PT was performed before the winter fallow period **(D)**, winter fallow period was bare fallow **(E,F)**, and RT was performed by rotavator before planting peanut **(G)**. **(H–K)** Represent green manure management (GM). Growing winter wheat after harvest of the peanut of the previous growing season **(H)**. Wheat plants were smashed by straw returning machine at the jointing stage **(I)**, and then plowed with a moldboard plow **(J)**, followed by the rotavator for the final seedbed preparation **(K)**.

Supplementary Figure 2 The application rate of green manure in 2016–2018 growing seasons.

Supplementary Figure 3 The LDA scores in each treatment from the LEfSe analysis. **(A)** 0–10 cm soil layer and **(B)** 10–20 cm soil layer.

Supplementary Figure 4 Box-plot histogram for Chao1 **(A,B)** and Shannon **(C,D)** indexes of the bacterial OTUs in 0–10 cm **(A,C)** and 10–20 cm **(B,D)** soil layer. Different letters indicate significant differences among each treatment, *P* < 0.05.

Supplementary Figure 5 Heatmap clustering analysis **(A,B)** and partial least square discriminant (PLS-DA) analysis **(C,D)** of the bacterial OTUs from treatments in 0–10 cm **(A,C)** and 10–20 cm **(B,D)** soil layer based on weighted UniFrac distances.

Supplementary Figure 6 Correlation coefficients between soil bulk density (SBD), soil porosity (SP), soil water content (SWC), soil organic carbon (SOC), available nitrogen (AN), available phosphorus (AP), available potassium (AK), urease, invertase, and catalase (CAT) activity. ^∗^Significant at the 0.05 probability level. ^∗∗^Significant at the 0.01 probability level.

## References

[B1] Akhtar K. Wang W. Y. Ren G. X. Khan A. Feng Y. Z. Yang G. H. (2018). Changes in soil enzymes, soil properties, and maize crop productivity under wheat straw mulching in Guanzhong. China. *Soil Till Res.* 182 94–102. 10.1016/j.still.2018.05.007

[B2] Baggs E. M. Watson C. A. Rees R. M. (2000). The fate of nitrogen from incorporated cover crop and green manure residues. *Nutr. Cycl Agroecosys* 56 153–163.

[B3] Bao S. D. (2000). *Soil and Agricultural Chemistry Analysis.* Beijing: Chinese Agriculture Press.

[B4] Bilalis D. Karkanis A. Efthimiadou A. Konstantas A. Triantafyllidis V. (2009). Effects of irrigation system and green manure on yield and nicotine content of Virginia (flue-cured) Organic tobacco (*Nicotiana tabaccum)*, under Mediterranean conditions. *Ind. Crop. Prod.* 29 388–394. 10.1016/j.indcrop.2008.07.007

[B5] Bogunovic I. Pereira P. Kisic I. Sajko K. Sraka M. (2018). Tillage management impacts on soil compaction, erosion and crop yield in Stagnosols (Croatia). *Catena* 160 376–384. 10.1016/j.catena.2017.10.009

[B6] Busari M. A. Kukal S. S. Kaur A. Bhatt R. Dulazi A. A. (2015). Conservation tillage impacts on soil crop and the environment. *Int. Soil Water Conserv. Res.* 3 119–129. 10.1016/j.iswcr.2015.05.002

[B7] Celik I. Ortas I. Kilic S. (2004). Effects of compost, mycorrhiza, manure and fertilizer on some physical properties of a Chromoxerert soil. *Soil Till Res.* 78 59–67. 10.1016/j.still.2004.02.012

[B8] Chatskikh D. Olesen J. E. (2007). Soil tillage enhanced CO2 and N2O emissions from loamy sand soil under spring barley. *Soil Till Res.* 97 5–18. 10.1016/j.still.2007.08.004

[B9] Chen H. Q. Liang Q. Gong Y. S. Kuzyakov Y. Fan M. S. Plante A. F. (2019). Reduced tillage and increased residue retention increase enzyme activity and carbon and nitrogen concentrations in soil particle size fractions in a long-term field experiment on Loess Plateau in China. *Soil Till Res.* 194:104296 10.1016/j.still.2019.104296

[B10] Chen S. Qi G. F. Luo T. Zhang H. C. Jiang Q. K. Wang R. (2017). Continuous-cropping tobacco caused variance of chemical properties and structure of bacterial network in soils. *Land. Degrad. Dev.* 29 4106–4120. 10.1002/ldr.3167

[B11] Chen W. Teng Y. Li Z. G. Liu W. X. Ren W. J. Luo Y. M. (2018). Mechanisms by which organic fertilizer and effective microbes mitigate peanut continuous cropping yield constraints in a red soil of south China. *Appl. Soil Ecol.* 128 23–34. 10.1016/j.apsoil.2018.03.018

[B12] Comerford N. (2005). “Soil factors affecting nutrient bioavailability,” in *Nutrient Acquisition by Plants. Ecological Studies (Analysis and Synthesis)*, Vol. 181 ed. BassiriRad H. (Berlin: Springer).

[B13] Couëdel A. Alletto L. Tribouillois H. Justes E. (2018). Cover crop crucifer-legume mixtures provide effective nitrate catch crop and nitrogen green manure ecosystem services. *Agr. Ecosyst. Environ.* 254 50–59. 10.1016/j.agee.2017.11.017

[B14] Dahal K. Knowles V. L. Plaxton W. C. Huner N. P. A. (2014). Enhancement of photosynthetic performance, water use efficiency and grain yield during long-term growth under elevated CO2 in wheat and rye is growth temperature and cultivar dependent. *Environ. Exp. Bot.* 106 207–220. 10.1016/j.envexpbot.2013.11.015

[B15] Das S. K. Varma A. (2011). “Role of enzymes in maintaining soil health,” in *Soil Enzymology*, eds Shukla G. Varma A. (Berlin: Springer-Verlag), 22–47.

[B16] Edgar R. C. (2013). UPARSE: highly accurate OTU sequences from microbial amplicon reads. *Nat. Methods* 10 996–998. 10.1038/nmeth.2604 23955772

[B17] Edgar R. C. Haas B. J. Clemente J. C. Quince C. Knight R. Notes A. (2011). UCHIME improves sensitivity and speed of chimera detection. *Bioinformatics* 27 2194–2200. 10.1093/bioinformatics/btr381 21700674 PMC3150044

[B18] Fan K. K. Delgado-Baquerizo M. Guo X. S. Wang D. Z. Zhu Y. G. Chu H. Y. (2020). Biodiversity of key-stone phylotypes determines crop production in a 4-decade fertilization experiment. *ISME J.* 10.1038/s41396-020-00796-8 [Epub ahead of print]. 33028975 PMC8027226

[B19] Gao H. H. Yan C. R. Liu Q. Ding W. L. Chen B. Q. Li Z. (2019). Effects of plastic mulching and plastic residue on agricultural production: a meta-analysis. *Sci. Total Environ.* 65 484–492. 10.1016/j.scitotenv.2018.09.105 30243168

[B20] Guan D. H. Al-kaisi M. M. Zhang Y. S. Duan L. S. Tan W. M. Zhang M. C. (2014). Tillage practices affect biomass and grain yield through regulating root growth, root-bleeding sap and nutrients uptake in summer maize. *Field Crop Res.* 157 89–97. 10.1016/j.fcr.2013.12.015

[B21] Guan S. Y. (1986). *Soil Enzymes and Research Methods. China Agricultural.* Beijing: Science Press.

[B22] Haas B. J. Gevers D. Ear A. M. Feldgarden M. Ward D. V. Giannoukos G. (2011). Chimeric 16S rRNA sequence formation and detection in Sanger and 454-pyrosequenced PCR amplicons. *Genome Res.* 21 494–504. 10.1101/gr.112730.110 21212162 PMC3044863

[B23] Han S. Li X. Luo X. S. Wen S. L. Chen W. L. Huang Q. Y. (2018). Nitrite-oxidizing bacteria community composition and diversity are influenced by fertilizer regimes, but are independent of the soil aggregate in acidic subtropical red soil. *Front. Microbiol.* 9:885. 10.3389/fmicb.2018.00885 29867799 PMC5951965

[B24] Haque M. M. Kim S. Y. Kim G. W. Kim P. J. (2015). Optimization of removal and recycling ratio of cover crop biomass using carbon balance to sustain soil organic carbon stocks in a mono-rice paddy system. *Agr. Ecosyst. Environ.* 207 119–125. 10.1016/j.agee.2015.03.022

[B25] Haro R. J. Dardanelli J. L. Otegui M. E. Collino D. J. (2008). Seed yield determination of peanut crops under water deficit: Soil strength effects on pod set, the source-sink ratio and radiation use efficiency. *Field Crop Res.* 109 24–33. 10.1016/j.fcr.2008.06.006

[B26] Huang F. Y. Liu Z. H. Mou H. Y. Li J. L. Zhang P. Jia Z. K. (2019). Impact of farmland mulching practices on the soil bacterial community structure in the semiarid area of the loess plateau in China. *Eur. J. Soil Biol.* 92 8–15. 10.1016/j.ejsobi.2019.04.001

[B27] Islam M. M. Urmi T. A. Rana M. S. Alam M. S. Haque M. M. (2018). Green manuring effects on crop morpho-physiological characters, rice yield and soil properties. *Physiol. Mol. Biol. Plants* 25 303–312. 10.1007/s12298-018-0624-2 30804651 PMC6352541

[B28] Kabiri V. Raiesi F. Ghazavi M. A. (2016). Tillage effects on soil microbial biomass, SOM mineralization and enzyme activity in a semi-arid Calcixerepts. *Agr. Ecosyst. Environ.* 232 73–84. 10.1016/j.agee.2016.07.022

[B29] Klindworth A. Pruesse E. Schweer T. Peplies J. Quast C. Horn M. (2013). Evaluation of general 16S ribosomal RNA gene PCR primers for classical and next-generation sequencing-based diversity studies. *Nucleic Acids Res.* 41:e1. 10.1093/nar/gks808 22933715 PMC3592464

[B30] Lee K. B. Liu C. T. Anzai Y. Kim H. Aono T. Oyaizu H. (2005). The hierarchical system of the ‘*Alphaproteobacteria’*: description of *Hyphomonadaceae* fam. nov., *Xanthobacteraceae* fam. nov. and *Erythrobacteraceae* fam. nov. *Int. J. Syst. Evol. Micr.* 55 1907–1919. 10.1099/ijs.0.63663-0 16166687

[B31] Li X. G. Ding C. F. Zhang T. L. Wang X. X. (2014). Fungal pathogen accumulation at the expense of plant-beneficial fungi as a consequence of consecutive peanut monoculturing. *Soil Biol. Biochem.* 72 11–18. 10.1016/j.soilbio.2014.01.019

[B32] Li Y. Y. Pang H. C. Han X. F. Yan S. W. Zhao Y. G. Wang J. (2016). Buried straw layer and plastic mulching increase microflora diversity in salinized soil. *J. Integr. Agr.* 15 1602–1611. 10.1016/s2095-3119(15)61242-4

[B33] Li Z. L. Tian D. S. Wang B. X. Wang J. S. Wang S. Chen H. Y. (2019). Microbes drive global soil nitrogen mineralization and availability. *Glob. Change Biol.* 25 1078–1088. 10.1111/gcb.14557 30589163

[B34] Lozupone C. Lladser M. E. Knights D. Stombaugh J. Knight R. (2011). UniFrac: an effective distance metric for microbial community comparison. *ISME J.* 5 169–172. 10.1038/ismej.2010.133 20827291 PMC3105689

[B35] Magoc T. Salzberg S. L. (2011). FLASH: fast length adjustment of short reads to improve genome assemblies. *Bioinformatics* 27 2957–2963. 10.1093/bioinformatics/btr507 21903629 PMC3198573

[B36] Miki T. Ushio M. Fukui S. Kondoh M. (2010). Functional diversity of microbial decomposers facilitates plant coexistence in a plant-microbe-soil feedback model. *PNAS* 107 14251–14256. 10.1073/pnas.0914281107 20663953 PMC2922608

[B37] Munger P. Bleiholder H. Hack H. Hess M. Stauss R. Van den Boom T. (1998). Phenological growth stages of the peanut plant (*Arachis hypogaea* L.) Codification and description according to the BBCH Scale – with figures. *J. Agron. Crop Sci.* 180 101–107. 10.1111/j.1439-037x.1998.tb00377.x

[B38] Muñoz-Rojas M. (2018). Soil quality indicators: critical tools in ecosystem restoration. *Curr. Opin. Environ. Sci. Health* 5 47–52. 10.1016/j.coesh.2018.04.007

[B39] Obermeier M. M. Minarsch E. M. L. Raj A. C. D. Rineau F. Schröder P. (2020). Changes of soil-rhizosphere microbiota after organic amendment application in a *Hordeum vulgare* L. short-term greenhouse experiment. *Plant Soil* 455 489–506. 10.1007/s11104-020-04637-7

[B40] Paul K. I. Polglase P. J. Oconnell A. M. Carlyle J. C. Smethurst P. J. Khanna P. K. (2003). Defining the relation between soil water content and net nitrogen mineralization. *Eur. J. Soil Sci.* 54 39–47. 10.1046/j.1365-2389.2003.00502.x

[B41] Pruesse E. Quast C. Knittel K. Fuchs B. M. Ludwig W. Peplies J. (2007). SILVA: a comprehensive online resource for quality checked and aligned ribosomal RNA sequence data compatible with ARB. *Nucleic Acids Res.* 35 7188–7196. 10.1093/nar/gkm864 17947321 PMC2175337

[B42] Puerta V. L. Oereira E. I. P. Wittwer R. Heijden M. Six J. (2018). Improvement of soil structure through organic crop management, conservation tillage and grass-clover ley. *Soil Till Res.* 180 1–9. 10.1016/j.still.2018.02.007

[B43] Qin S. H. Yeboah S. Xu X. X. Liu Y. H. Yu B. (2017). Analysis on fungal diversity in rhizosphere soil of continuous cropping potato subjected to different furrow-ridge mulching managements. *Front. Microbiol.* 8:845. 10.3389/fmicb.2017.00845 28539923 PMC5423957

[B44] Raiesi F. Kabiri V. (2017). Carbon and nitrogen mineralization kinetics as affected by tillage systems in a calcareous loam soil. *Ecol. Eng.* 106 24–34. 10.1016/j.ecoleng.2017.05.023

[B45] Reeves D. W. (1997). The role of soil organic matter in maintaining soil quality in continuous cropping systems. *Soil Till Res.* 43 131–167. 10.1016/s0167-1987(97)00038-x

[B46] Reicosky D. C. (2015). Conservation tillage is not conservation agriculture. *J. Soil Water Conserv.* 70 103–108.

[B47] Schloss P. D. Westcott S. L. Ryabin T. Hall J. R. Hartmann M. Hollister E. B. (2009). Introducing mothur: open-source, platform-independent, community-supported software for describing and comparing microbial communities. *Appl. Environ. Microbiol.* 75 7537–7541. 10.1128/aem.01541-09 19801464 PMC2786419

[B48] Sengupta A. Dick W. A. (2015). Bacterial community diversity in soil under two tillage practives as determined by pyrosequencing. *Microb. Ecol.* 70 853–859. 10.1007/s00248-015-0609-4 25930203

[B49] Sharma P. Laor Y. Raviv M. Medina S. Saadi I. Krasnovsky A. (2017). Green manure as part of organic management cycle: effects on changes in organic matter characteristics across the soil profile. *Geoderma* 305 197–207. 10.1016/j.geoderma.2017.06.003

[B50] Shen P. Wu Z. F. Wang C. X. Luo S. Zheng Y. M. Yu T. Y. (2016). Contributions of rational soil tillage to compaction stress in main peanut producing areas of China. *Sci. Rep.U. K.* 6:38629. 10.1038/srep38629 PMC5146654 27934905

[B51] Shen Y. F. Chen Y. Y. Li S. Q. (2016). Microbial functional diversity, biomass and activity as affected by soil surface mulching in a semiarid farmland. *PLoS One* 11:e1059144. 10.1371/journal.pone.0159144 27414400 PMC4945083

[B52] Shi M. Sun Y. Y. Wang Z. H. Gang H. Quan H. X. He H. X. (2019). Plastic film mulching increased the accumulation and human health risks of phthalate esters in wheat grains. *Environ. Pollut.* 250 1–7. 10.1016/j.envpol.2019.03.064 30981178

[B53] Shi Y. Yu Z. W. Man J. G. Ma S. Y. Gao Z. Q. Zhang Y. L. (2016). Tillage practices affect dry matter accumulation and yield in winter wheat in the North China Plain. *Soil Tillage Res.* 160 73–81. 10.1016/j.still.2016.02.009

[B54] Šibanc N. Dumbrell A. J. Mandić-Mulecd I. Mačekab I. (2014). Impacts of naturally elevated soil CO2 concentrations on communities of soil archaea and bacteria. *Soil Biol. Biochem*, 68 348–356. 10.1016/j.soilbio.2013.10.018

[B55] Subrahmaniyan K. Veeramani P. Harisudan C. (2018). Heat accumulation and soil properties as affected by transparent plastic mulch in Blackgram (*Vigna mungo*) doubled cropped with Groundnut (*Arachis hypogaea*) in sequence under rainfed conditions in Tamil Nadu. India. *Field Crop Res.* 219 43–54. 10.1016/j.fcr.2018.01.024

[B56] Sul W. J. Asuming-Brempong S. Wang Q. Tourlousse D. M. Penton C. R. Deng Y. (2013). Tropical agricultural land management influences on soil microbial communities through its effect on soil organic carbon. *Soil Biol. Biochem.* 65 33–38. 10.1016/j.soilbio.2013.05.007

[B57] Sun T. Li G. Ning T. Y. Zhang Z. M. Mi Q. H. Lal R. (2018). Suitability of mulching with biodegradable film to moderate soil temperature and moisture and to increase photosynthesis and yield in peanut. *Agric. Water Manag.* 208 214–223. 10.1016/j.agwat.2018.06.027

[B58] Takaichi S. Maoka T. Takasaki K. Hanada S. (2010). Carotenoids of *Gemmatimonas aurantiaca* (*Gemmatimonadetes*): identification of a novel carotenoid, deoxyoscillol 2-rhamnoside, and proposed biosynthetic pathway of oscillol 2,2′-dirhamnoside. *Microbiol* 156 757–763. 10.1099/mic.0.034249-0 19959572

[B59] Talgre L. Lauringson E. Roostalu H. Astover A. Makke A. (2012). Green manure as a nutrient source for succeeding crops. *Plant Soil Environ.* 58 275–281. 10.17221/22/2012-pse

[B60] Tao J. M. Liu X. D. Liang Y. L. Niu J. J. Xiao Y. H. Gu Y. B. (2017). Maize growth responses to soil microbes and soil properties after fertilization with different green manures. *Appl. Microbiol. Biot.* 101 1289–1299. 10.1007/s00253-016-7938-1 27816989

[B61] Tian S. Z. Ning T. Y. Wang Y. Liu Z. Li G. Li Z. J. (2016). Crop yield and soil carbon responses to tillage method changes in North China. *Soil Till Res.* 163 207–213. 10.1016/j.still.2016.06.005

[B62] Trivedi P. Delgado-Baquerizo M. Anderson I. C. Singh B. K. (2016). Response of soil properties and microbial communities to agriculture: implications for primary productivity and soil health indicators. *Front. Plant Sci.* 7:990. 10.3389/fpls.2016.00990 27462326 PMC4940416

[B63] Tyler H. L. (2019). Bacterial community composition under long-term reduced tillage and no till management. *J. Appl. Microbiol.* 126 1797–1807. 10.1111/jam.14267 30925202

[B64] Vaz C. M. P. Bassoi L. H. Hopmans J. W. (2001). Contribution of water content and bulk density to field soil penetration resistance as measured by a combined cone penetrometer- TDR probe. *Soil Till Res.* 60 35–42. 10.1016/s0167-1987(01)00173-8

[B65] Wacal C. Ogata N. Basalirwa D. Sasagawa D. Ishigaki T. Handa T. (2019). Imbalanced soil chemical properties and mineral nutrition in relation to growth and yield decline of sesame on different continuously cropped upland fields converted paddy. *Agronomy* 9:184 10.3390/agronomy9040184

[B66] Wang C. Liu D. W. Bai E. (2018). Decreasing soil microbial diversity is associated with decreasing microbial biomass under nitrogen addition. *Soil Biol. Biochem.* 120 126–133. 10.1016/j.soilbio.2018.02.003

[B67] Wang Q. Garrity G. M. Tiedje J. M. Cole J. R. (2011). Naive bayesian classifier for rapid assignment of rRNA sequences into the new bacterial taxonomy. *Appl. Environ. Microbiol.* 73 5261–5267. 10.1128/aem.00062-07 17586664 PMC1950982

[B68] Wang Z. T. Liu L. Chen Q. Wen X. X. Liao Y. C. (2016). Conservation tillage increases soil bacterial diversity in the dryland of northern China. *Agron. Sustain. Dev.* 36:28.

[B69] Yang Y. H. Ding J. L. Zhang Y. H. Wu J. C. Zhang J. M. Pan X. Y. (2018). Effects of tillage and mulching measures on soil moisture and temperature, photosynthetic characteristics and yield of winter wheat. *Agric. Water Manag.* 201 299–308. 10.1016/j.agwat.2017.11.003

[B70] Yao G. (2004). *Peanut Production and Utilization in the People’s Republic of China.* Peanut in Local and Global Food Systems Series Report No. 4. Athens, GA: University of Georgia, 26.

[B71] Zhang D. B. Yao P. W. Zhao N. Yu C. W. Cao W. D. Gao Y. J. (2016). Contribution of green manure legumes to nitrogen dynamics in traditional winter wheat cropping system in the Loess Plateau of China. *Eur. J. Agron.* 72 47–55. 10.1016/j.eja.2015.09.012

[B72] Zhang H. Sekiguchi Y. Hanada S. Hugenholtz P. Kim H. Kamagata Y. (2003). *Gemmatimonas aurantiaca* gen. nov., sp. nov., a Gram-negative, aerobic, polyphosphate-accumulating micro-organism, the first cultured representative of the new bacterial phylum *Gemmatimonadetes phyl*. nov. *Int. J. Syst. Evol. Micr* 53 1155–1163. 10.1099/ijs.0.02520-0 12892144

[B73] Zhang X. X. Zhang R. J. Gao J. S. Wang X. C. Fan F. L. Ma X. T. (2017). Thirty-one year of rice-rice-green manure rotations shape the rhizosphere microbial community and enrich beneficial bacteria. *Soil Biol. Biochem.* 104 208–217. 10.1016/j.soilbio.2016.10.023

[B74] Zhang F. Zhang W. J. Li M. Zhang Y. Li F. M. Li C. B. (2017a). Is crop biomass and soil carbon storage sustainable with long-term application of full plastic film mulching under future climate change? *Agric. Syst.* 150 67–77. 10.1016/j.agsy.2016.10.011

[B75] Zhang F. Zhang W. J. Li M. Yang Y. S. Li F. M. (2017b). Does long-term plastic film mulching really decrease sequestration of organic carbon in soil in the Loess Plateau? *Eur. J. Agron.* 89 53–60. 10.1016/j.eja.2017.06.007

[B76] Zhang M. Cheng G. Feng H. Sun B. H. Zhao Y. Chen H. X. (2017). Effects of straw and biochar amendments on aggregate stability, soil organic carbon, and enzyme activities in the Loess Plateau. China. *Environ. Sci. Pollut. R* 24 10108–10120. 10.1007/s11356-017-8505-8 28233202

[B77] Zhou G. X. Zhang J. B. Zhang C. Z. Feng Y. Z. Chen L. Yu Z. H. (2016). Effects of changes in straw chemical properties and alkaline soils on bacterial communities engaged in straw decomposition at different temperatures. *Sci. Rep.* 6:22186. 10.1038/srep22186 PMC4768159 26916902

[B78] Zuber S. M. Villamil M. B. (2016). Meta-analysis approach to assess effect of tillage on microbial biomass and enzyme activities. *Soil Biol. Biochem.* 97 176–187. 10.1016/j.soilbio.2016.03.011

