# Supplementary material for: Effects of Soil Tillage, Management Practices, and Mulching Film Application on Soil Health and Peanut Yield in a Continuous Cropping System
Source: Front Microbiol. 2020 Dec 23;11:570924. doi: 10.3389/fmicb.2020.570924 (PMC7785755; doi:10.3389/fmicb.2020.570924)
Supplement: Supplementary Figure 1 — Different soil tillage and management practices applied to the experimental field. (A–C) Represent reduced tillage management. Winter fallow period was bare fallow and no tillage was performed during the period (A,B), and rotary tillage (RT) was adopted before planting peanut (C). (D–G) Represent conventional tillage management consisting of plow tillage (PT) and RT. PT was performed before the winter fallow period (D), winter fallow period was bare fallow (E,F), and RT was performed by rotavator before planting peanut (G). (H–K) Represent green manure management (GM). Growing winter wheat after harvest of the peanut of the previous growing season (H). Wheat plants were smashed by straw returning machine at the jointing stage (I), and then plowed with a moldboard plow (J), followed by the rotavator for the final seedbed preparation (K). [file Presentation_1.pdf]

Supplementary figure

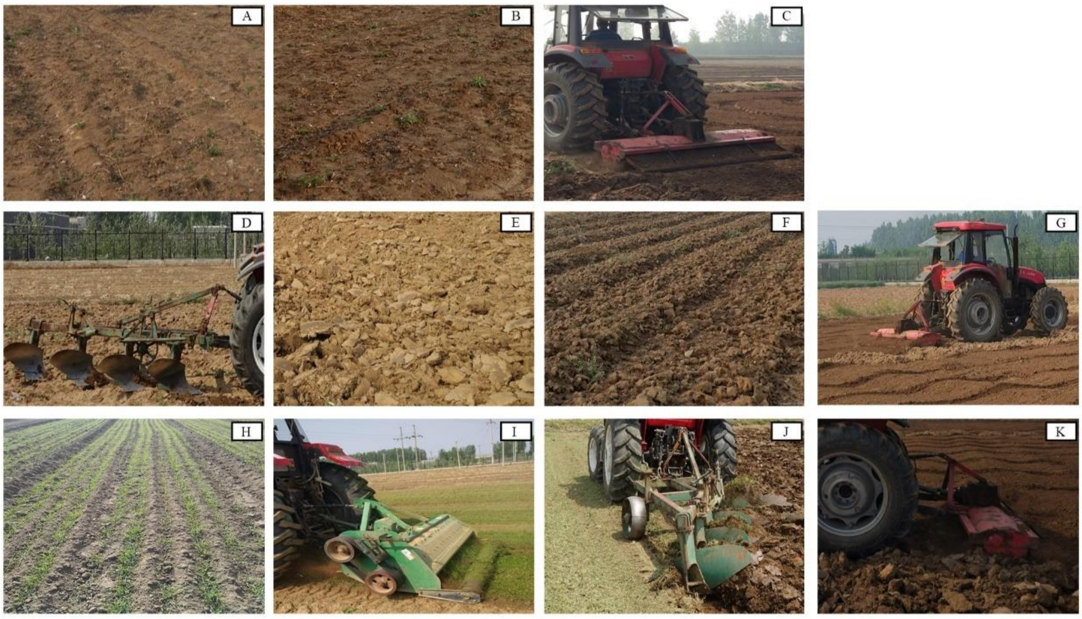

Supplementary Figure 1

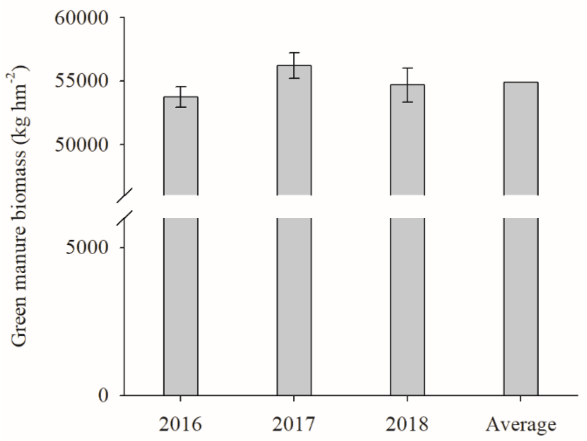

Supplementary Figure 2

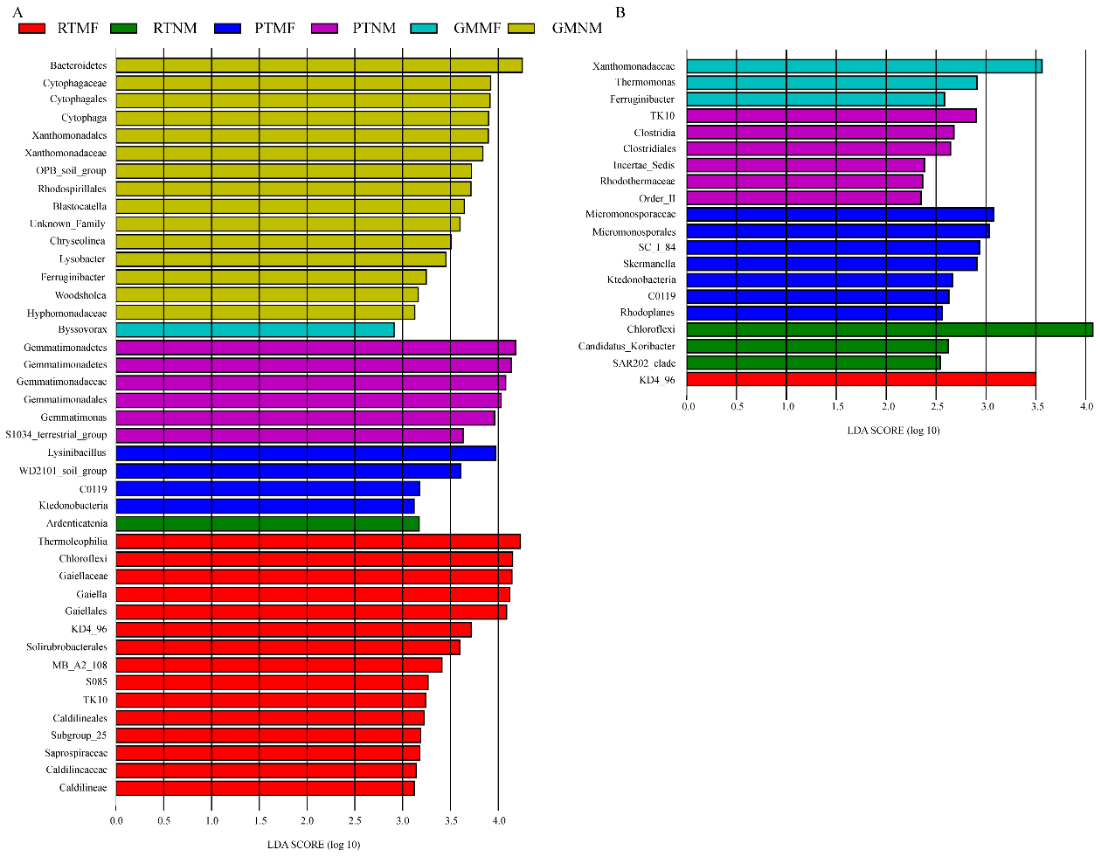

Supplementary Figure 3

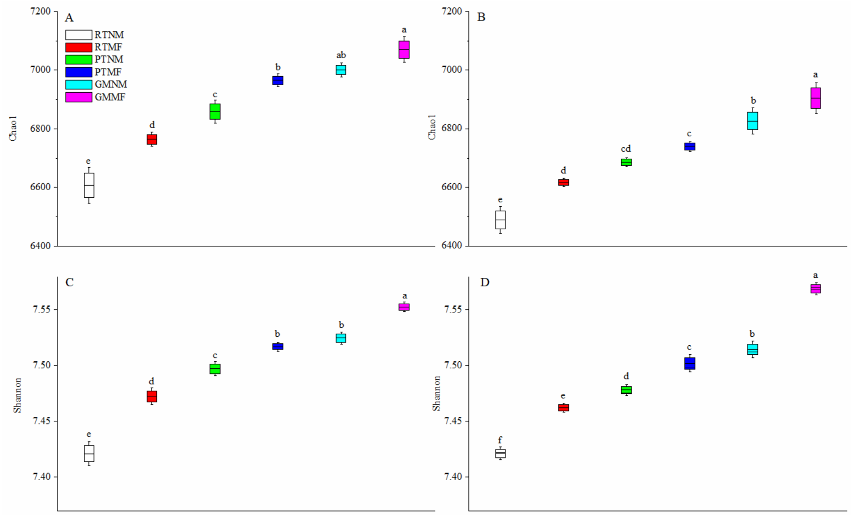

Supplementary Figure 4

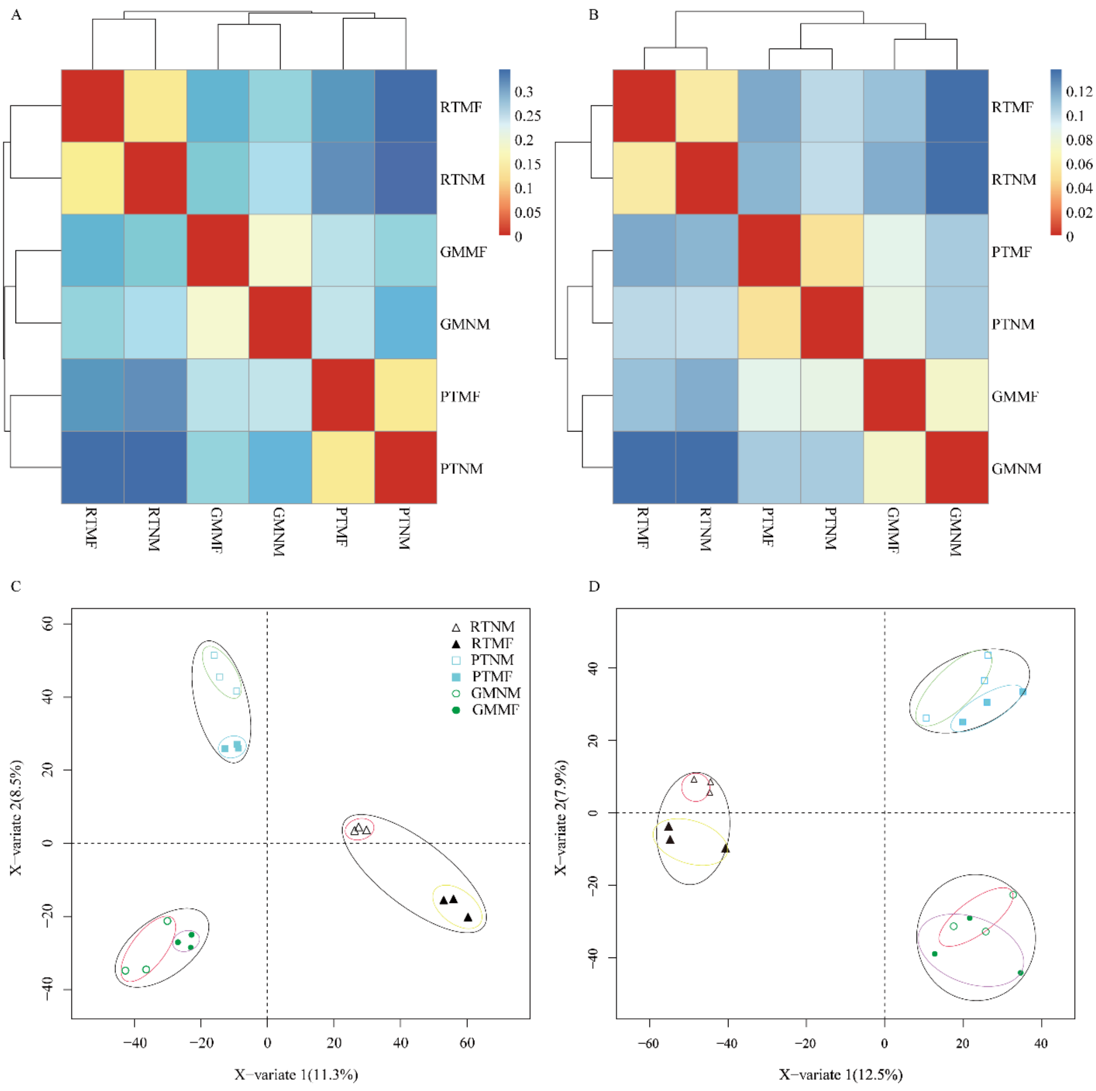

Supplementary Figure 5

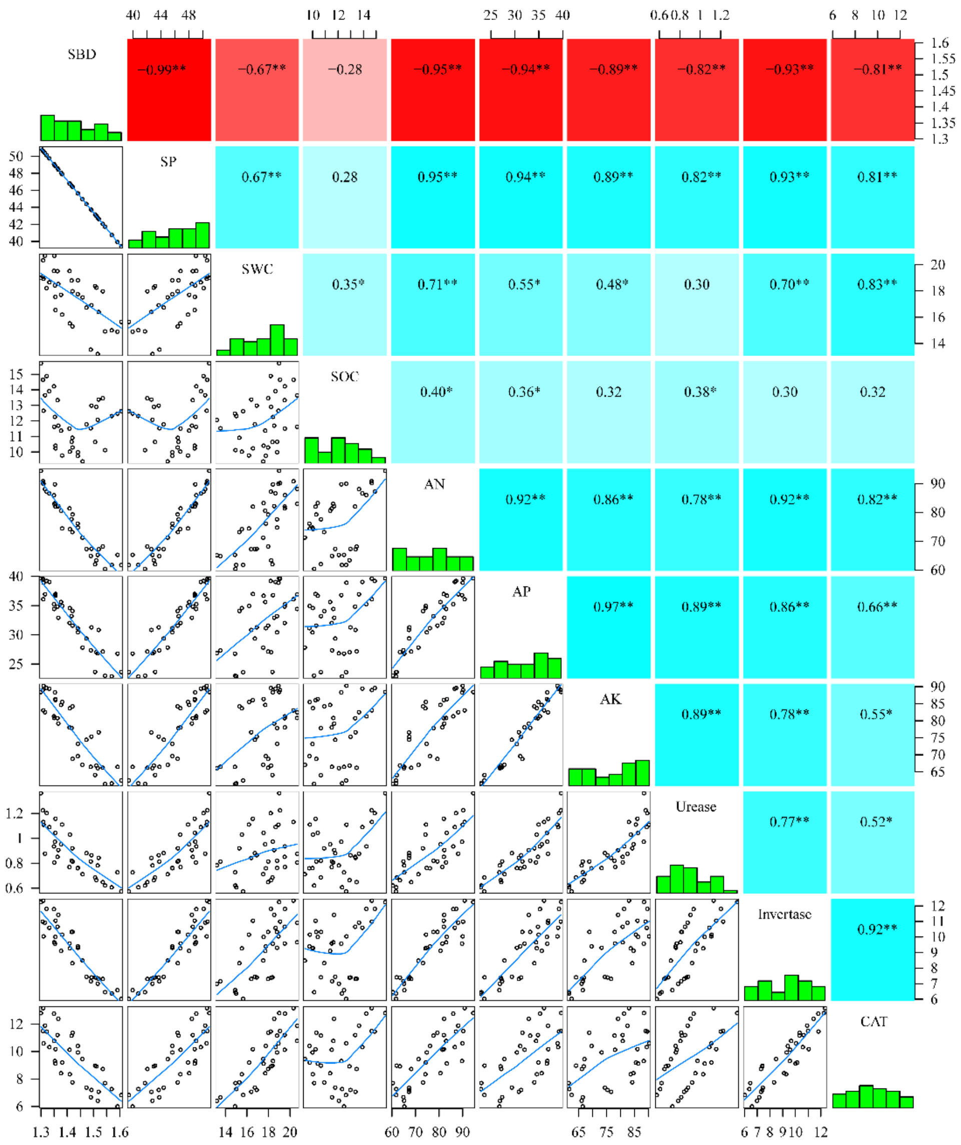

Supplementary Figure 6
